# Endoscopic submucosal dissection using a novel multi traction device

**DOI:** 10.1055/a-2702-4948

**Published:** 2025-10-13

**Authors:** Sarah Leblanc, Thomas Lambin, Bertrand Napoleon, Vincent Lepilliez

**Affiliations:** 1 Digestive Endoscopy Unit, Hôpital Privé Jean Mermoz, Ramsay-Santé, Lyon, France

Endoscopic submucosal dissection (ESD) is considered the recommended technique by the European Society of Gastrointestinal Endoscopy (ESGE) for the treatment of colorectal lesions greater than 2 cm, especially those presenting risks of limited submucosal invasion 1 . To facilitate ESD, various traction techniques have been described. The combination of a clip and orthodontic elastic is commonly used 2 . Recently, a new European Conformity (CE)-marked traction device has become available. This system consists of a clip attached to four aligned rubber rings allowing multipoint traction during ESD. This device has not been previously described in the literature; here we present a case report illustrating its use.

The case involves a 41-year-old woman with a 6-cm non-granular laterally spreading tumour located in the transverse colon. The lesion was classified as Paris IIa, Kudo Vi, Sano IIIa ( Fig. 1 ). Due to the presence of a non-granular component and lesion size, resection via ESD was selected, employing the novel traction device ( Video 1 ). The system was introduced through the operating channel and rotated to facilitate precise positioning. Initially, the clip connected to the traction rings was applied to one edge of the lesion. Then, a hemostatic clip secured the distal ring to the opposite edge ( Fig. 2 ). Finally, a third clip provided traction by attaching one of the central rings to the opposite colonic wall, exposing the submucosal space widely for optimal dissection ( Fig. 3 ). At the end of the procedure, the lesion was retrieved by pulling a free ring with coagulation forceps. The lesion was removed after a 60-minute procedure, yielding a specimen measuring 60 × 40 mm ( Fig. 4 ). Pathology demonstrated an adenoma with high grade dysplasia with R0 margins, confirming curative resection.

**Fig. 1:**
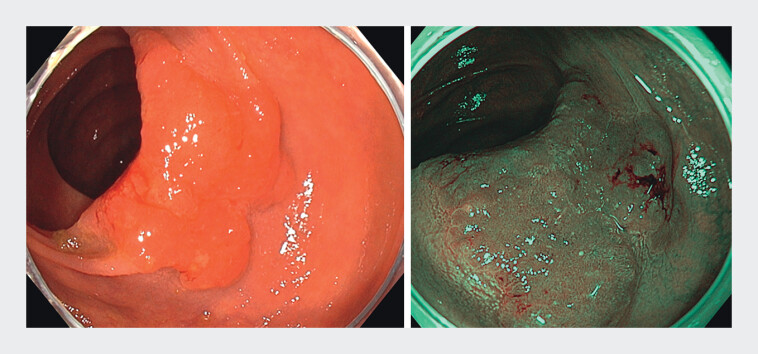
A 6-cm non-granular laterally spreading tumour located in the transverse colon (left: white light imaging; right: narrow-band imaging).

Endoscopic submucosal dissection using a novel multipoint traction device.Video 1

**Fig. 2:**
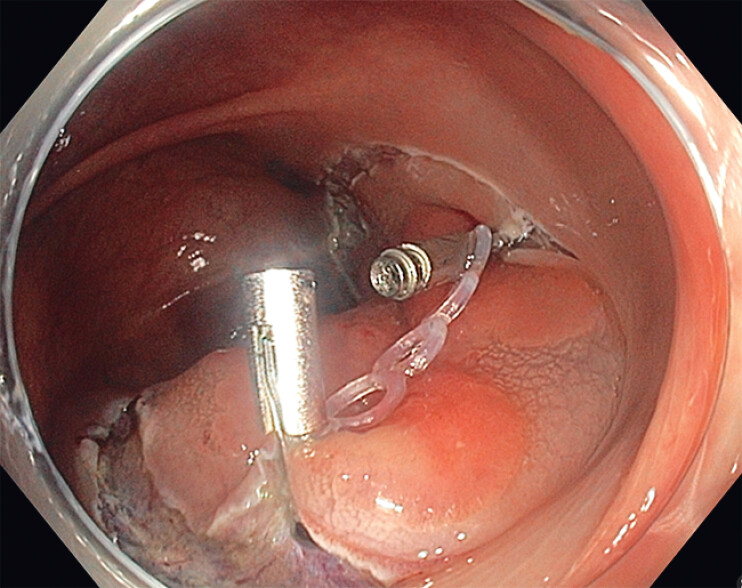
The traction device attached to the two edges of the lesion.

**Fig. 3:**
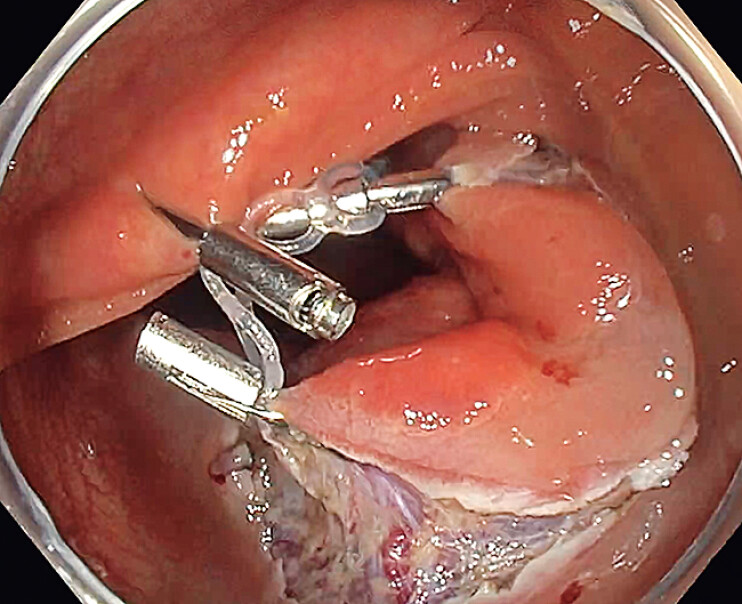
The device attached to the opposite colonic wall to provide sufficient traction.

**Fig. 4:**
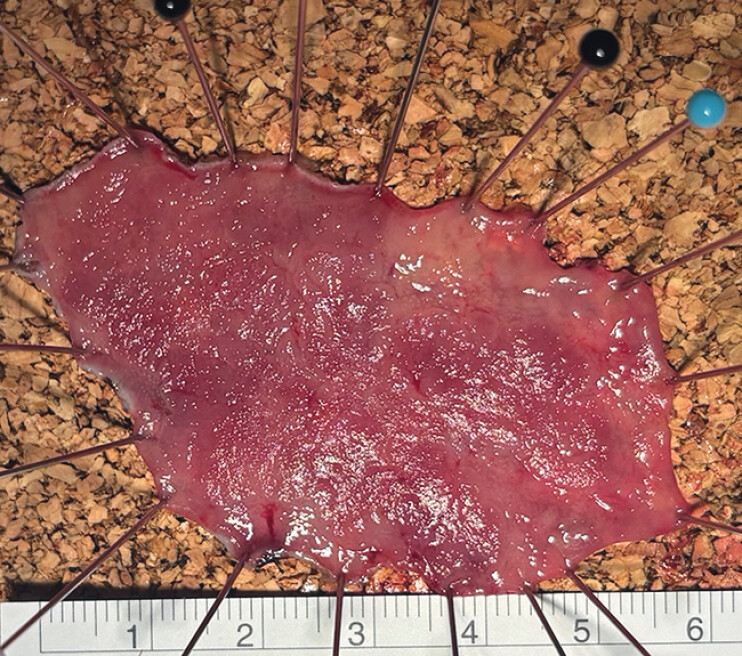
The lesion was removed after a 60-minute procedure, yielding a specimen measuring 60×40 mm.

This new through-the-scope traction device allows multipoint and stable traction with wide submucosal exposure. Further studies are warranted to compare its efficacy with existing devices.

Endoscopy_UCTN_Code_TTT_1AQ_2AD_3AD

## Notes

Endoscopy E-Videos https://eref.thieme.de/e-videos
*E-Videos* is an open access online section of the journal *Endoscopy* , reporting on interesting cases and new techniques in gastroenterological endoscopy. All papers include a high-quality video and are published with a Creative Commons CC-BY license. Endoscopy E-Videos qualify for HINARI discounts and waivers and eligibility is automatically checked during the submission process. We grant 100% waivers to articles whose corresponding authors are based in Group A countries and 50% waivers to those who are based in Group B countries as classified by Research4Life (see: https://www.research4life.org/access/eligibility/ ). This section has its own submission website at https://mc.manuscriptcentral.com/e-videos .
